# Effects of Population Size on Synchronous Display of Female and Male Flowers and Reproductive Output in Two Monoecious *Sagittaria* Species

**DOI:** 10.1371/journal.pone.0048731

**Published:** 2012-10-31

**Authors:** Xiufang Wang, Wen Zhou, Jing Lu, Haibin Wang, Chan Xiao, Jing Xia, Guihua Liu

**Affiliations:** 1 Key Laboratory of Aquatic Botany and Watershed Ecology, Wuhan Botanical Garden, the Chinese Academy of Sciences, Wuhan, China; 2 Graduate School of the Chinese Academy of Sciences, Beijing, China; University of Konstanz, Germany

## Abstract

**Background:**

Flowering synchrony and floral sex ratio have the potential to influence the mating opportunities and reproductive success through female function. Here, we examine the variances in synchronous display of female and male function, ratio of male to female flowers per day and subsequently reproductive output in small populations of two monoecious plants, *Sagittaria trifolia* and *Sagittaria graminea.*

**Methodology/Principal Finding:**

We created plant populations of size 2, 4, 10 and 20 and recorded the daily number of blooming male and female flowers per plant to determine daily floral display, flowering synchrony index and ratio of male to female flowers per day. We also harvested the fruits, counted the seeds and calculated the number of fruits and seeds per flower to measure reproductive success through female function. There is less overlap in flowering time of female and male function in smaller populations than in larger populations. Most importantly, we found that male-biased floral sex ratio and imbalanced display period of female and male function for individual plant can lead to a population-size-dependent ratio of male to female flowers per day. Increasing ratio of male to female flowers per day was generally associated with a greater percentage of fruit production.

**Conclusions/Significance:**

Our results highlight the importance of flowering synchrony of female and male function and population-size-dependent ratio of male to female flowers per day for female reproductive success. This finding improves our understanding of a mechanism that reduces reproductive success in small populations.

## Introduction

Flowering synchrony has the potential to influence the mating opportunities and reproductive success [1], [2]. Theoretical studies have predicted that reproductive asynchrony, which occurs when males and females are reproductively active at different times, could increase reproductive failure as the probability of temporal overlap of potential mates, especially in small populations, is reduced [3]–[5]. It is easy to understand that for animals, when populations are at a reduced size or density, individuals may face difficulties in finding mates. Similarly, a female flower may have fewer occasions to be pollinated in a small population where it is less likely that another plant will be in male-phase simultaneously.

In monoecious populations, the floral sex ratio also has the potential to affect mating opportunities. Pollinator-meditated selection should result in larger male floral displays and hence higher ratio of male to female flowers, because the number of male flowers influences pollen production and attraction to pollinators [6]–[9]. On the other hand, sexual selection should favor extended duration of male function, thus increasing the number and variety of mating partners [10]–[15]. Studies investigating flower dimorphism and floral display with unisexual flowers (monoecy or dioecy) often report male function extends the duration of their floral displays through producing or opening only a fraction of their flowers each day compared to female function [10], [11], [16]. Hence, the functional significance of floral display is not the total number of flowers produced by an inflorescence or ramet but rather the number of flowers in anthesis each day [11]. Although the floral sex ratio for individuals or populations of monoecious species may be relatively less variable in common garden experiments due to similar plant size and environmental conditions, if the floral display of female and male function in an individual is not constant through time, the number and relative ratio of synchronous open female and male flowers may be associated with population size at a given time. To assess the above possibility, we need to examine the sex-specific daily floral production.

*Sagittaria* (Alismataceae), a worldwide genus of emergent aquatics with monoecious and dioecious sexual systems [10], [16], is a good system to study how interactions among population size, flowering synchrony, and ratio of male to female flowers per day affect reproductive success. Individual plants of the monoecious *Sagittaria* species can produce several sequential racemes. Female flowers occur on basal whorls of the inflorescence and male flowers on upper whorls. It is infrequent for flowering periods of female and male function to overlap within an inflorescence [10], [16]–[18]; thus, monoecious *Sagittaria* are protogynous at the inflorescence level. The mating system of *Sagittaria* species is dominated by outcrossing mechanisms [19], [20]. This means that the monoecious *Sagittaria* species are temporally dioecious and there must be synchrony between two sexual flowers of different ramets for reproductive purposes. In addition, each ramet of the *Sagittaria* species typically has more male flowers than female flowers, and individual flowers last a single day. For a given ramet, female flowers open more synchronously, whereas the flowering period of male flowers is more gradual with male function lasting much longer [21], [22].

**Table 1:** Effects of population size and floral sex on the floral display size, the total number of days that flowers bloomed for ramets and for populations in *S. trifolia* and *S. graminea* analysed with mixed model ANOVA.

		Floral display size		Total number of flowering days for a ramet		Total number of flowering days for a population	
Source	d.f.	*F*	*P*	*F*	*P*	*F*	*P*
*S. trifolia*							
Floral sex	1,211	61560.07	0.00	1273.70	0.00	224.66	0.00
Population size	3,211	1.38	0.34	1.24	0.38	126.24	0.00
*S. graminea*							
Floral sex	1,211	93832.32	0.00	1927.77	0.00	156.89	0.00
Population size	3,211	0.18	0.91	2.34	0.18	72.97	0.00

In this paper, we investigated the relation of population size, daily floral display, flowering synchrony and female reproductive success in two related *Sagittaria* species (a native *S. trifolia* and an exotic *S. graminea*) using experimental manipulation of the number of individuals per population. We addressed the following questions: (i) What are the patterns of variation in daily floral display for female and male function among population sizes? (ii) What is the relationship between the flowering synchrony of female and male functions (measured by synchrony index and ratio of male to female flowers per day) and population sizes? (iii) What is the relationship between female reproductive success (measured by seed output) and ratio of male to female flowers per day? and (iv) How does the population size affect seed production?

**Figure 1:**
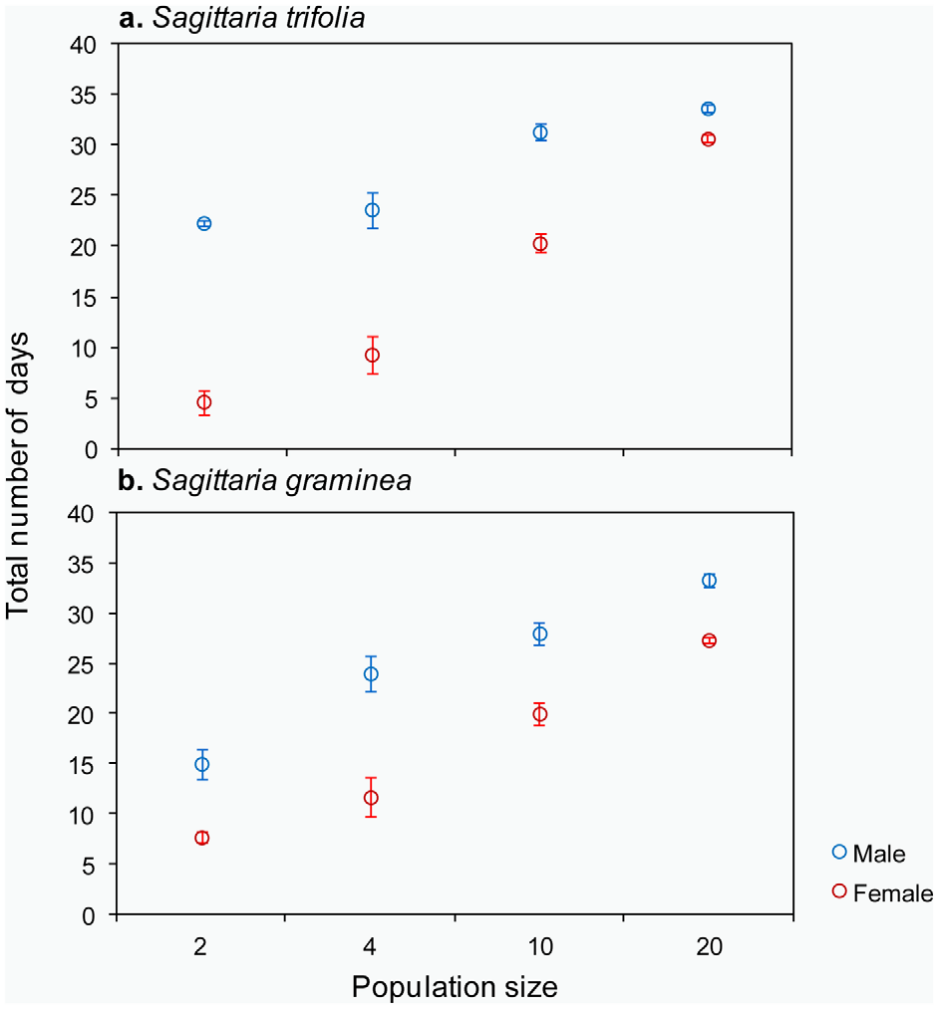
The mean (± SE) of the total number of days that male and female flowers were open for different population sizes of *S. trifolia* and *S. graminea*.

## Materials and Methods

### Study species

*Sagittaria trifolia* (Alismataceae) is an emergent aquatic plant that grows in a variety of wetland habitats, including the edges of ponds, small rivers, irrigation ditches and rice fields in Asia. Plants reproduce both sexually and clonally by producing shoot tubers. Unisexual, nectar-producing flowers are visited by a wide spectrum of pollinators including bees, flies, beetles, butterflies and wasps, and individual flowers last a single day [10]. Female flowers are smaller than males. The plant is originally a common weed in paddy fields. However, populations of *S. trifolia* have diminished and become isolated as herbicide use became more widespread in China. Small populations with fewer than 20 ramets occur widely in natural conditions. *Sagittaria graminea* is native to North America and was introduced to China in the 1990s as a floriculture species for gardens. *Sagittaria trifolia* differs from *S. graminea* in possessing larger inflorescences and more flowers.

**Figure 2:**
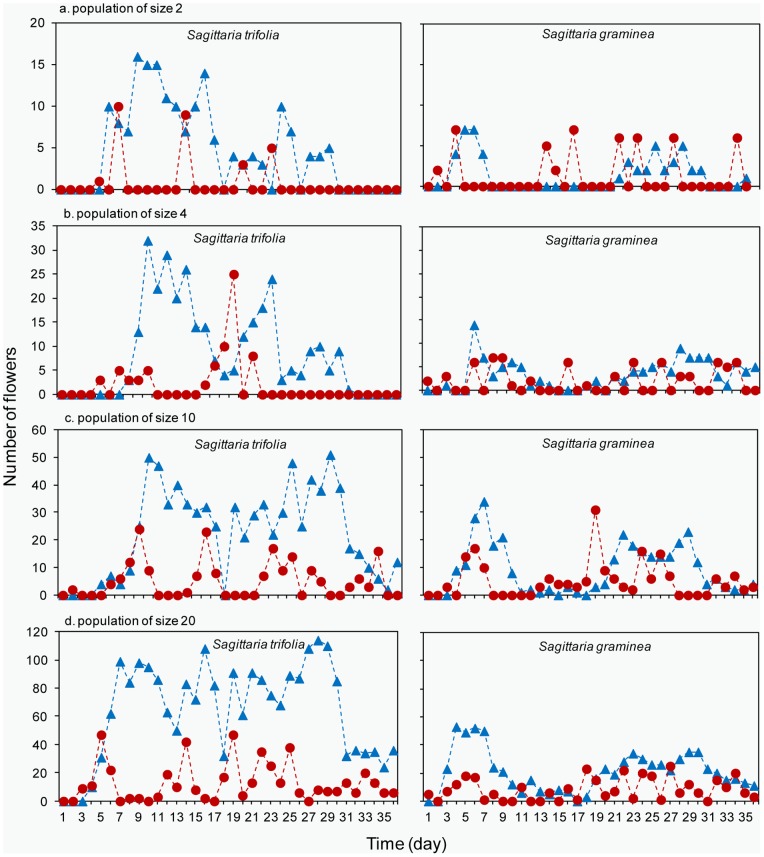
Daily display size of male (blue triangle) and female flowers (red circle) of *S. trifolia* and *S. graminea* using representative examples from population of four different sizes (a) 2, (b) 4, (c) 10 and (d) 20, respectively.

### Experimental design

This study was conducted at the Wuhan Botanical Garden, China, in 2007. We manipulated the population sizes by setting up arrays of potted plants of *S. trifolia* and *S. graminea*. Seeds of *S. trifolia* and *S. graminea* were collected from 20 plants from the same source population at the Wuhan Botanical Garden in 2006 and were germinated in the laboratory during the spring of 2007. Individual seedlings were transplanted into separate pots (20 cm diameter, one seedling per pot) with some natural topsoil. The pots were kept in a greenhouse and watered daily until flowering began. Flowering plants were assigned at random to create synthetic populations (plots) of size 2, 4, 10 and 20 in the Wuhan Botanical Garden. The entire experimental design was repeated three times. In total, 108 plants were used to construct the 12 experimental populations for each species. Plants within populations were placed 0.75 m apart from their nearest neighbors. Neighboring populations were separated by at least 100 m, a distance that had previously been found to substantially reduce pollen flow among populations of *S. trifolia* [22]. Populations were grown in the field for 34 days to allow natural insect pollination. Most of the surrounding landscape was composed of sparse seedlings from shrubs.

**Figure 3:**
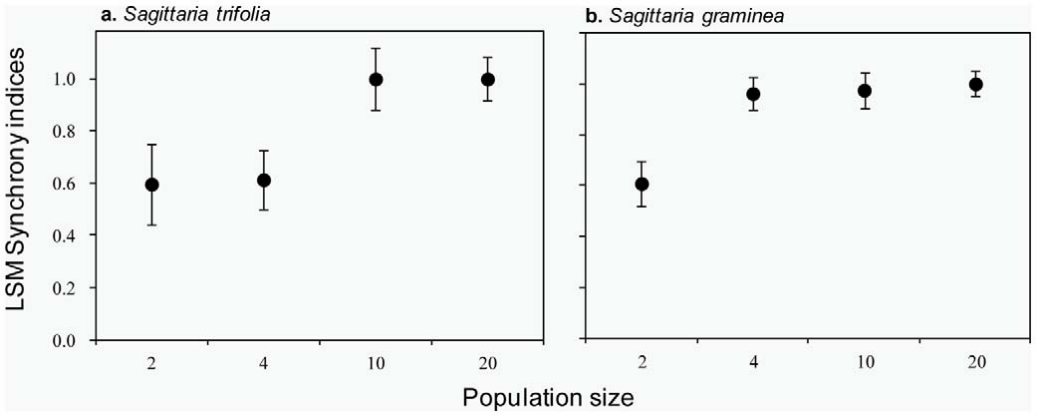
The mean (± SE) of flowering synchrony indices within populations of (a) *S. trifolia* and (b) *S. graminea*.

### Floral display

To examine the variance in the floral display size, the total number of days that flowers bloomed for each ramet and for experimental populations, we counted daily the number of open male and female flowers per ramet. We used two-way analysis of variance to examine whether the total number of flowering days varied among population sizes and between floral sexes. A mixed-model analysis of variance with three main factors (floral sex [fixed], population size [fixed], and plot [random]) was used to assess the effects of floral sex and population size on floral display and the total number of flowering days for ramet [23]. The response variables were transformed by log_10_ to meet test assumptions.

**Figure 4:**
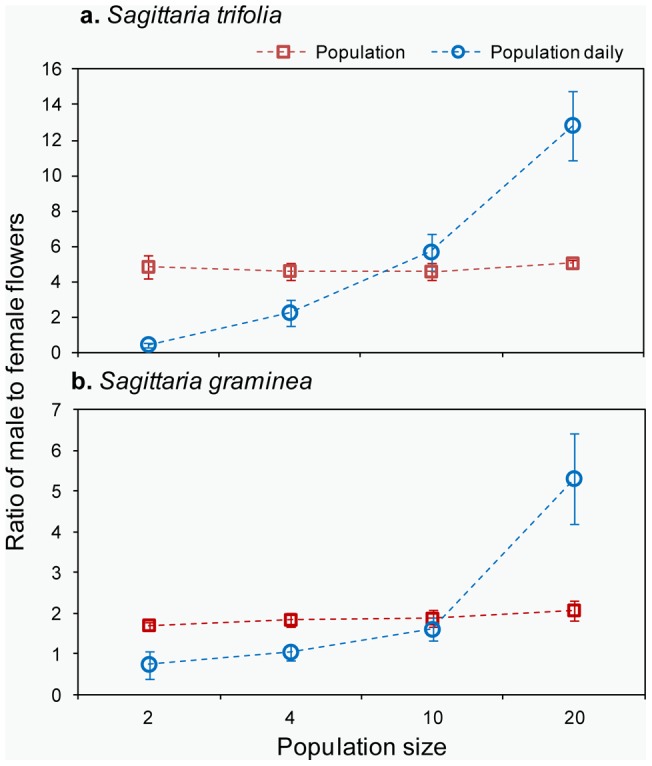
The mean population floral sex ratio and ratio of male to female flowers per day (± SE) in different population sizes of *S. trifolia* and *S. graminea* (*n* = 108).

A synchrony index has been developed to measure flowering overlap of female and male flowers within populations, which is calculated using the following formula [24]:
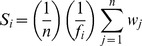
where *S_i_* is the synchrony index for a ramet *i*, *n* is the number of male flowering plants within a population, i.e. *n* = 2, 4, 10 and 20 in four manipulated population sizes, respectively, *f_i_* is the number of census days for female flower blooming of ramet *i*, and *w_j_* is the number of census days for female flowers of plant *i* and male flowers of plant *j* blooming simultaneously. The synchrony indices were analyzed with a mixed-model analysis of variance. Population size was used as a fixed factor and plot as a random factor. Values of *S_i_* were transformed by arcsin square root to meet test assumptions.

**Figure 5:**
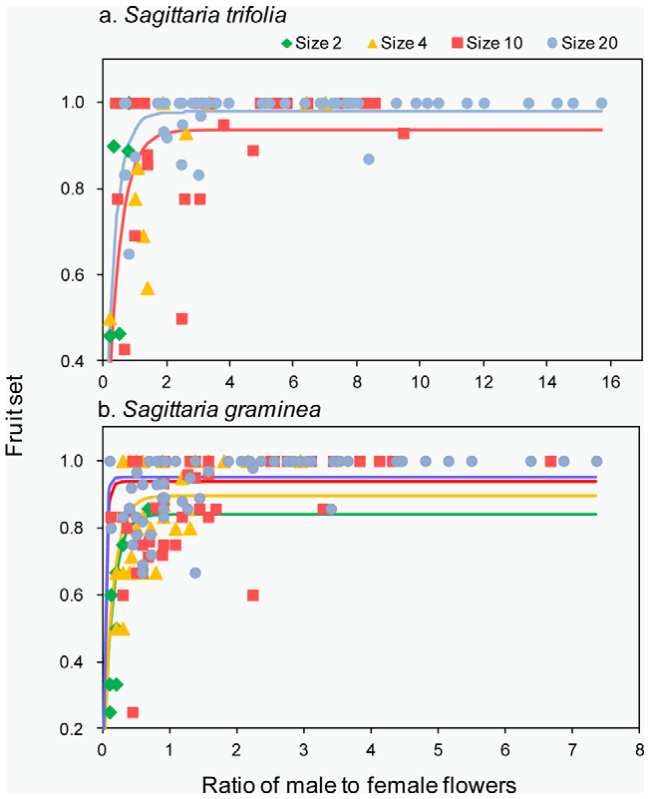
Dose-response relationships between the proportion of flowers setting fruit and the intra-day ratio of male-to-female flowers in (a) *S. trifolia* and (b) *S. graminea*. The equations were: (a) population of size 10, Y = 0.94(1-e^−2.45X^), *r* ^2^ = 0.96, *P*<0.001; population of size 20, Y = 0.98(1-e^−3.15X^), *r* ^2^ = 0.98, *P*<0.001; (b) population of size 2, Y = 0.84(1-e^−4.38X^), *r* ^2^ = 0.98, *P*<0.001; population of size 4, Y = 0.89(1-e^−4.22X^), *r* ^2^ = 0.97, *P*<0.001; population of size 10, Y = 0.94(1-e^−5.58X^), *r* ^2^ = 0.96, *P*<0.001; population of size 20, Y = 0.95(1-e^−7.98X^), *r* ^2^ = 0.99, *P*<0.001. No significant relationships were detected for populations of size 2 and 4 in *S. trifolia*.

### Floral sex ratio and fruit and seed production

To determine the effect of population size on flowering pattern of female and male function, we investigated the variation in ramet and population floral sex ratio, as well as the ratio of male to female flowers per population per day. The ramet and population floral sex ratio were calculated by dividing the total number of male flowers by the total number of female flowers per ramet or per population during the entire experiment, respectively. The ratio of male to female flowers per day was used as a measurement for daily male to female function production, since female reproductive success primarily depends on the relative quantity of synchronously open male flowers. The ratio of male to female flowers per day was calculated by dividing the number of open male flowers by the number of open female flowers for each population per day. When only single sexual flowers are open the pollination cannot really be performed and thus those values were excluded from the calculation. We used a mixed-model analysis of variance to investigate the effects of population size on the three sex ratios. Population size was a fixed factor whereas plot was random.

**Figure 6:**
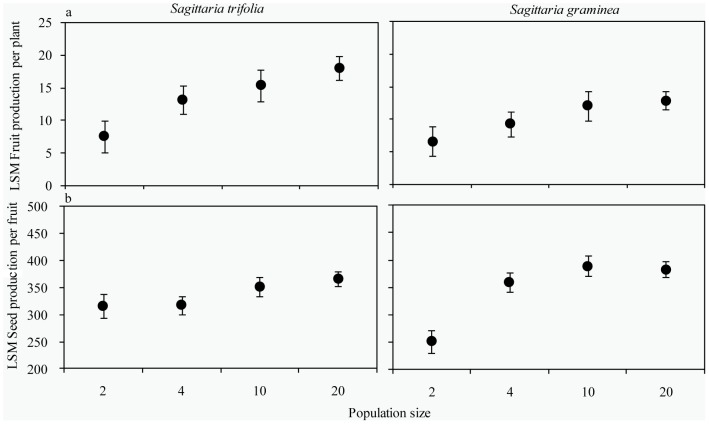
Reproductive success of *S. trifolia* and *S. graminea*. (a) Least-square means (LSM) for total fruit production per plant, (b) LSM for seed production per fruit in populations of different sizes. Bars represent confidence limits to 95%.

At the end of the experiment, we harvested all aggregate fruits (henceforth called fruit), and counted mature seeds. To determine whether fruit production related to the ratio of male to female flowers per day within populations, the blooming dates of female flowers were recorded on a daily basis. Fruit set was estimated as the proportions of flowers that developed into fruits for each population per day. Thus, fruit sets correspond with their same-day-recorded ratio of male to female flowers per day. Since fruit set should be a saturating function of the ratio of male to female flowers per day, we examined the non-linear dose–response relationship between the sex ratio and the fruit set for each population size, using nonlinear regression (procedure NLIN, Marquart estimation) to fit data to the negative exponential function:
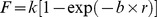
where *k* estimates the maximum fruit set and *b* defines the rate at which the asymptote is approached [25]. The fruit and seed production per plant was analysed using generalized linear mixed models with a Poisson distribution and a logarithmic link function. We used population size as a fixed factor and plot as random factor.

## Results

### Floral display

No significant differences in inflorescence production were detected among population sizes in *S. trifolia* (ranging from 1.9 to 2.2 inflorescences, *χ* ^2^ = 0.63, df  = 3, *P*>0.05) and in *S. graminea* (ranging from 2.5 to 2.9 inflorescences, *χ* ^2^ = 0.65, df  = 3, *P*>0.05). More male flowers (*S. trifolia*: 79.11±2.41; *S. graminea*: 32.87±0.63) opened than female flowers (*S. trifolia*: 18.81±0.59; *S. graminea*: 15.70±0.48) for each ramet (Table 1). The flowering period per plant was significantly different between the floral sexes (Table 1), with more days of blooming in male flowers (*S. trifolia*: 19.19±0.38; *S. graminea*: 14.63±0.30) than female flowers (*S. trifolia*: 3.17±0.12; *S. graminea*: 3.94±0.17). No significant effects were observed for population size on floral display size and flowering period in ramet (Table 1).

On a population level, the total number of flowering days were significantly different among population sizes and floral sexes in both *S. trifolia* and *S. graminea* (Table 1): the total number of open days for male flowers lasted significantly longer than that for female flowers in each population size, and the total number of open days in larger populations lasted significantly longer than in smaller populations for each floral sex (Fig. 1).

In *S. trifolia*, the proportion of days on which male flowers were present were 62%, 71%, 88% and 100%, while those for female flowers were 15%, 29%, 59% and 91% in population sizes of 2, 4, 10 and 20, respectively (Fig. 2). Similarly in *S. graminea*, the proportion of flowering days of males were 47%, 71%, 76% and 97%, while those of female flowers were 24%, 47%, 65% and 82% with respect to the already mentioned population sizes (Fig. 2). The ratios of male to female flowers per day in *S. graminea* were more variable than those in *S. trifolia*.

Both *S. trifolia* and *S. graminea* showed a significantly greater flowering synchrony with increasing population size (Fig. 3. *S. trifolia*: *F*_3, 104_ = 68.03, *P*<0.01; *S. graminea*: *F*_3, 104_ = 34.56, *P*<0.01). In *S. trifolia*, the overlap between female and male function were 60% and 61% in populations of size 2 and 4, which is significantly smaller than 100% of flowering overlap in populations of size 10 and 20. Similarly, in *S. graminea*, a flowering overlap of 61% in 2-plant populations was significantly smaller than the 96%, 98% and 100% flowering overlap seen in the three other population sizes.

### Sex-ratio variation and reproductive success

At the individual level, there were no significant differences in the floral sex ratio per ramet among population sizes in *S. trifolia* (ranging from 4.1 to 4.4, *F*_3, 104_ = 0.47, *P*>0.05) and in *S. graminea* (ranging from 1.7 to 2.3, *F*_3, 104_ = 1.85, *P*>0.05). The mean total floral sex ratios of individual plants were 4.21±0.27 and 2.09±0.18 in *S. trifolia* and in *S. graminea*, respectively.

At the population level, there were no significant differences in the population floral sex ratios among population sizes in *S. trifolia* (*F*_3,8_ = 0.47, *P*>0.05, Fig. 4a) and in *S. graminea* (*F*_3,8_ = 1.88, *P*>0.05, Fig. 4b). However, we detected a significantly increased ratio of male to female flowers per day with increasing population size (*S. trifolia*: *F*_3, 89_ = 75.92, *P*<0.01; *S. graminea*: *F*_3, 89_ = 46.84, *P*<0.01. Fig. 4).

Nonlinear dose-response examination indicates that the fruit set was significantly influenced by the ratio of male to female flowers per day (Fig. 5). A much higher proportion of flowers developed into fruits with an increasing ratio of male to female flowers per day for each population size in both *S. trifolia* and *S. graminea*. The asymptotic fruit set in smaller populations fell noticeably below that in larger populations.

The fruit production per plant was significantly different among population sizes in both *S. trifolia* (*χ* ^2^ = 54.72, df  = 3, *P*<0.01, Fig. 6a) and *S. graminea* (*χ* ^2^ = 48.66, df  = 3, *P*<0.01). Similarly, the seed production per fruit was also significantly different among population sizes in both *S. trifolia* (*χ* ^2^ = 86.78, df  = 3, *P*<0.01, Fig. 6b) and *S. graminea* (*χ* ^2^ = 125.32, df  = 3, *P*<0.01).

## Discussion

We detected that the ramets of the two *Sagittaria* species produced significantly more male flowers than female flowers. Each ramet opened only a fraction of their male flowers at once and thereby extended their floral display duration, while female function opened more synchronously and thereby performed a larger daily floral display. Consistent with our predictions, we found that population size can significantly influence synchronous display of female and male function and the ratio of male to female flowers per day. We also detected that increasing ratios of male to female flowers per day were generally associated with a greater fruit and seed production.

At the ramet level, we detected male-biased floral sex ratios in the two monoecious species *S. trifolia* and *S. graminea*. A similar pattern is also observed in the monoecious species *S. brevirostra* [17] and *S. sagittifolia* [18] and *S. latifolia* [11], and is consistent with the widely held expectation that selection to increase mating opportunities should favor larger male production and greater ratio of male to female flowers [10]–[15]. On the other hand, our observations in *S. trifolia* and *S. graminea* also indicate that only a few male flowers are in bloom at any given time in comparison to female function, leading to male function displays over a longer period of time. Such pattern has been previously reported in several *Sagittaria* species, including *S. trifolia* [10], [11], [16], [26]. Most studies have emphasized that this pattern might be expected to increase male mating opportunities [10]–[15].

It should be noted, however, that extended exposure of male function might also reduce the ratio of male to female flowers in anthesis and thereby increase the likelihood of pollen limitation. For example, a ramet of *S. trifolia* produces on average 79 male flowers in 19 days and 19 female flowers is 3 days, and thus opens about 4 male flowers and 6 female flowers per day, respectively. This leads to a lower ratio of male to female flowers of about 0.67. Studies have demonstrated that flowering sex ratios can influence the degree of pollen limitation of females in gender-dimorphic species [1]–[3]. As expected, we found a lower ratio of male to female flowers per day in small population, but the ratios significantly increased with the increasing population sizes, although the overall population sex ratio over the season is not significantly affected by population size. Previous studies in monoecious species have attributed the variation of floral sex ratio among populations to the quality of environmental condition [27]–[30] and plant size [17], [18], [26], [31]. In the present paper, the effects of environment and plant size are unlikely to be responsible for the observed differences in the ratio of male to female flowers per day. Firstly, experimental ramets originated from the same batch of seedlings, and were grown under similar environmental conditions. Secodly, there was no significant difference in the floral sex ratio per ramet among population sizes.

Variation of flowering synchrony between female and male function and demographic stochasticity may contribute to population-size-dependent variation in the ratio of male to female flowers per day. Firstly, the likelihood that anthesis of male and female flowers overlaps is lower in small populations than in large populations due to demographic stochasticity [32], [33]. Secondly, because the male function of ramets lasts much longer than the female function, at the population level, it is more likely that one ramet's male function period overlaps with that of another ramet much more so than would occur with two ramets' female function periods. For example, female function lasts on average 3 d whereas male function is completed in 19 d, in *S. trifolia*. This means that in a flowering season of 36 d, for example, at the very least, twelve females but only two males must flower to cover the length of the flowering season, assuming that same-sex flowers do not have overlapping flowering periods. This pattern leads to quicker accumulation of synchronously open male flowers than female flowers in larger populations. Finally, because the female flowers always open first for each ramet of the two studied species [10], [21], the earliest-opening female flowers may exhibit greater effects to population daily flowering sex ratio in small populations than in larger ones.

In the two studied monoecious species, the ratio of male to female flowers per day appears to have a strong impact on female reproductive success, which is consistent with previous conclusions in gynodioecious species [1.2] and in a theoretical model [3]. First, evidence from pollination experiments on *S. trifolia* and *S. latifolia* support the inference that pollinators of *Sagittaria* species respond strongly to floral display size [9], [10]. Secondly, because the frequency of male flowers determines the availability of pollen, the ratio of male to female flowers per day can affect the degree to which female flowers are pollen limited [2], [34]–[36]. Finally, because the flowering times of male and female flowers are completely random, demographic stochasticity might result in increased deviations in flowering overlap between male and female flowers, especially in very small populations [32], [33]. In addition, it should further be noted that ratios of male to female flowers of about 4 in *S. trifolia* and about 2 in *S. graminea* are sufficient to yield full seed set, suggesting highly male-biased populations are inefficient.
